# Implantable Thermal Therapeutic Device with Precise Temperature Control Enabled by Foldable Electronics and Heat-Insulating Pads

**DOI:** 10.34133/2022/9787296

**Published:** 2022-05-24

**Authors:** Min Cai, Huang Yang, Liyin Shen, Shuang Nie, Zhengwei Mao, Changyou Gao, Yang Zhu, Jizhou Song

**Affiliations:** ^1^Department of Rehabilitation Medicine, the First Affiliated Hospital College of Medicine, Zhejiang University, Hangzhou 310003, China; ^2^Department of Engineering Mechanics, Soft Matter Research Center, and Key Laboratory of Soft Machines and Smart Devices of Zhejiang Province, Zhejiang University, Hangzhou 310027, China; ^3^MOE Key Laboratory of Macromolecular Synthesis and Functionalization, Department of Polymer Science and Engineering, Zhejiang University, Hangzhou 310027, China; ^4^State Key Laboratory of Fluid Power and Mechatronic Systems, Zhejiang University, Hangzhou 310027, China

## Abstract

Thermal therapy has continued to attract the attention of researchers and clinicians due to its important applications in tumor ablation, wound management, and drug release. The lack of precise temperature control capability in traditional thermal treatment may cause the decrease of therapeutic effect and thermal damage to normal tissues. Here, we report an implantable thermal therapeutic device (ITTD), which offers precise closed loop heating, in situ temperature monitoring, and thermal protection. The ITTD features a multifunctional foldable electronics device wrapped on a heat-insulating composite pad. Experimental and numerical studies reveal the fundamental aspects of the design, fabrication, and operation of the ITTD. In vivo experiments of the ITTD in thermal ablation for antitumor demonstrate that the proposed ITTD is capable of controlling the ablation temperature precisely in real time with a precision of at least 0.7°C and providing effective thermal protection to normal tissues. This proof-of-concept research creates a promising route to develop ITTD with precise temperature control capability, which is highly desired in thermal therapy and other disease diagnosis and treatments.

## 1. Introduction

Thermal therapy has continued to attract the attention of researchers and clinicians because of its important applications in tumor ablation [1, 2], wound management [3, 4], and drug release [5]. To achieve the best therapeutic outcomes, the temperature should be controlled precisely during heating since underheating or overheating will induce undesired results of the decrease of therapeutic effect and the thermal damage of normal tissues close to the lesion target [6]. For example, when the target heating area is adjacent to large blood vessels, the thermal therapeutic effect will be significantly reduced because the heating temperature cannot rise in time due to the loss of much heat energy carried away by blood vessels. Traditional thermal treatments by the methods of photothermal [7, 8], magnetothermal [9, 10], ultrasound [11], or radiofrequency [12, 13], have shown great promise in practical applications, but none can provide the precise temperature control capability due to the lack of real-time and in situ temperature monitoring. During their operations, the temperature of heating area can quickly rise above threshold to induce thermal damages to normal tissues. An ideal thermal therapeutic device for thermal therapy should be able to heat the target and monitor the temperature simultaneously for precise temperature control to optimize the therapeutic effect and prevent damaging adjacent tissues. The recent advances of flexible electronics with features of self-powering [14], intrinsically stretchability [15], and integrating of optoelectronic function [16, 17] allow them to be worn on or implanted in bodies and provide opportunities to revolutionize the medical devices for health monitoring [18, 19], diagnosis [20, 21], and therapy [22–25]. However, the integration of real-time and in situ heating/sensing mechanism with thermal protection functionality into a single thermal therapeutic device with simple structures still remains a challenge.

Here, we report a novel implantable thermal therapeutic device (ITTD), which offers the capabilities of precise closed loop heating, in situ temperature monitoring, and thermal protection to optimize the therapeutic effect while preventing thermal damage to adjacent normal tissues. The ITTD features a multifunctional foldable electronic device with delicate designs of heater and temperature sensor wrapped on a heat-insulating composite pad with hollow glass microspheres mixed in a grooved polydimethylsiloxane (PDMS) substrate. The foldability of the multifunctional flexible electronics consisting of an in situ pair of heater/sensor and an isolated temperature sensor facilitates the simple device configuration and simplifies the fabrication processes of ITTD. The grooved heat-insulating composite pad could limit the thermal diffusion direction, causing a large temperature difference between the target tissue and normal tissue, thus improving the heating efficiency on target tissue and protecting the adjacent normal tissue from thermal damage. Experimental and numerical studies reveal the fundamental aspects of the closed loop heating, in situ temperature sensing, and thermal protection. In vivo tumor thermal ablation experiments demonstrate that the proposed ITTD is capable of monitoring and adjusting the ablation temperature precisely in real time, detecting the temperature of adjacent tissue, and providing the effective thermal protection. Our ITTD with precise temperature control capability offers an alternative to the minimally invasive thermal treatment techniques (e.g., microwave and RF ablation), which cannot easily address the problems of large area heating, normal tissue thermal protection, and complex equipment requirements. The proposed ITTD could potentially complement available thermal treatment techniques and narrow the gap between the noninvasive thermal treatmet methods and the minimally invasive methods.

## 2. Results

### 2.1. Design of the Implantable Thermal Therapeutic Device

**Figure 1:**
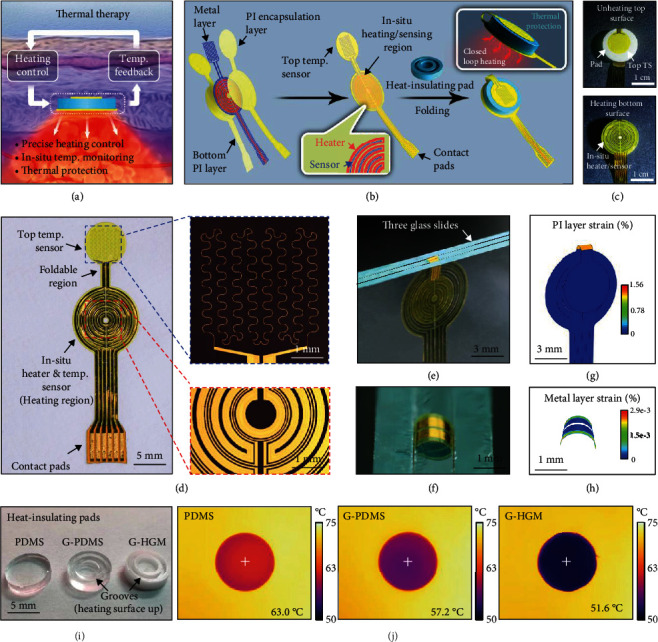
Design concept, layout, and optical images of the implantable thermal therapeutic device (ITTD). (a) Desired features of an ideal ITTD for thermal therapy. (b) Design concept of the ITTD with precise temperature control capability enabled by a multifunctional foldable electronic device wrapped on a heating-insulating composite pad. (c) Optical images of (top) the unheating surface and (bottom) heating surface of the ITTD. (d) Optical and microscopic images of the multifunctional foldable electronic device consisting of an in situ heater/temperature sensor and a top temperature sensor. (e, f) Optical image of the folded device with a close-up view. (g, h) Maximum principal strain profiles in external PI layer and inner metal layer. (i) Optical image of the heat-insulating pads. (j) Thermal images of the unheating surfaces of the PDMS, G-PDMS, and G-HGM heat-insulating pads.

Figure 1(a) schematically shows the features of the proposed implantable thermal therapeutic device (ITTD) with the capabilities of precise closed loop heating, in situ temperature monitoring, and thermal protection, which are highly desired in practical applications of tumor ablation, would management, and drug release. As illustrated in Figure 1(b), the ITTD is composed of a multifunctional foldable thin film electronic device wrapped on a grooved heat-insulating composite pad. The schematic in Figure 1(b) (left) shows the tilted exploded view layout of the multifunctional foldable thin film electronic device constructed by a layer of metal (Cr/Au, 5/150 nm in thickness) sandwiched by the patterned polyimide (PI) films (14 *μ*m in thickness) for insulation and protection. The detailed design of the multifunctional foldable electronics is shown in Figure S1. A quarter of the actual heating region on Figure 1(b) (middle inset) clearly shows the delicate in situ design of the heater (red traces) and temperature sensor (blue traces). This delicate in situ heater/sensor architecture enable the real-time and in situ temperature monitoring and provides the significant feedback information for precise temperature control, analysis of heating effect in real time, and the precaution of tissue-overheating damage. The folding of the foldable electronic device covers the top and bottom surfaces of the heat-insulating composite pad and yields the final ITTD (Figure 1(b), right). This construct simultaneously enables the bottom surface of the pad to provide the precise closed loop heating and the top surface to monitor temperature for preventing the overheating damage of the normal tissue (Figure 1(b), right inset). Figure 1(c) shows optical images of the unheating top surface and the heating bottom surface of the fully device, respectively.

Figure 1(d) shows the optical image of the foldable electronic device with a blue dotted box showing the microscopic image of the serpentine structure of top temperature sensor (top TS) and the red one showing the microscopic image of the traces of the in situ heater (265 *μ*m in line width) and temperature sensor (in situ TS) (35 *μ*m in line width). The contact pads are designed to link the homemade adaptor for the external power and data acquisition. The foldable region connects the top TS and the heating region (11 mm in diameter). To demonstrate the excellent foldability, we fold the foldable region to form a concentric circular structure of the top TS and the heating region through three pieces of glass slides for clamping and fixing (Figure 1(e)) with the foldable region shown by a close-up view (Figure 1(f)), reaching 0.6 mm in the radius of curvature. As demonstrated by finite element analysis (FEA) results (Figures 1(g) and 1(h), the maximum principal strains in the external PI layer (1.56%) and the inner metal layer (0.0029%) are both much less than their damage limits (9.8% and 1%, respectively) [26, 27], which ensures the function of heater and sensor during the folding process.

To achieve a better heat-insulating performance, we introduced grooves on the heating surface of pad to enhance the interfacial thermal resistance and mix hollow glass microspheres in the pad to enhance the material thermal resistance (i.e., decrease the thermal conductivity) as well. To illustrate the benefits of these design concepts, three kinds of heat-insulating pads were fabricated (Figure 1(i)). The first is the soft polydimethylsiloxane pad (PDMS, 2.5 mm in thickness and 10.5 mm in diameter) without any design considerations. The second is the grooved PDMS pad (G-PDMS) with grooves on the heating surface. The third is the grooved PDMS composite pad (G-HGM) by mixing the hollow glass microspheres (HGM) (lower thermal conductivity, <0.1 W m^−1^ K^−1^) in the PDMS precursor. The design details and fabrication of the PDMS pad, the G-PDMS pad, and the G-HGM pad are descried in Figure S2. The thermal conductivity of PDMS composite pads doped with 0-20% *w*/*w* hollow glass microspheres is shown in Figure S3, which clearly indicates the benefit of decreasing the thermal conductivity.

For simple comparisons of the heat-insulating performance, these three types of pads are placed on a 75°C heating stage with the grooved side directly contacting to the stage. The temperature on the top surface (unheating surface) rises due to the heat flux passing through the interfacial surface and the pad material. Because of the differences of these three pads' thermal resistances, the heat-insulating performance has a significant distinction. As illustrated in the steady-state temperature distributions recorded by a thermal imager (Figure 1(j)), the maximum temperatures on the unheating surfaces of the PDMS pad, the G-PDMS pad, and the G-HGM pad are 63°C, 57.2°C, and 51.6°C, respectively. As expected, the G-HGM pad with design considerations has the best heat-insulating performance.

### 2.2. Performance Characterization of the ITTD

**Figure 2:**
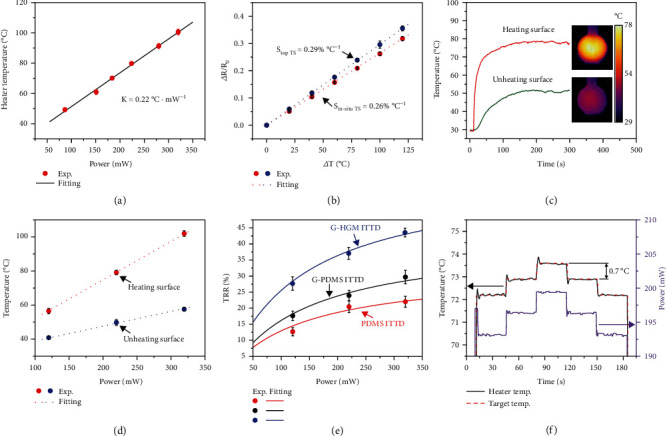
Characterization of heating, monitoring, and heat-insulting performances of ITTD. (a) The heating temperature as the function of applied heating power. (b) Relative electrical resistance changes of the top and in situ temperature sensors under various temperature changes. (c) Temperature curves of the heating surface and the unheating surface at 220 mW with the corresponded thermal images embedded. (d) Steady temperatures of the heating surface and unheating surface at various applied heating powers. (e) Temperature reduction rate (TTR) of the PDMS, G-PDMS, and G-HGM ITTDs at different powers. (f) Temperature adjustment curves with a small change of 0.7°C.

Systematic experimental studies were carried out to investigate the heating, monitoring, and heat-insulating performances of the ITTD with a G-HGM pad.  Figure 2(a) shows the heating performance of heater by describing the relationship between the heating temperature (measured by the thermal imager) and the power applied on the heater. The heater exhibits a good linearity (*K* = 0.22°C mW^−1^, *R*^2^ = 0.99) in a broad heating range up to 102°C, which covers most of the required thermal therapeutic temperatures [28]. This data shows the heating performance of heater in the air environment. The heating performances in other environments were not investigated because the tissue temperature is not obtained from this heating performance but from the in situ temperature sensor, which allows us to adjust the heating power in real time. Thus, predicting the heating performances in different environments is not necessary.  Figure 2(b) describes the temperature sensing performances of the top TS and the in situ TS and gives the relationship between the electrical resistance variation ratio (Δ*R*/*R*_0_ with Δ*R* as the measured change in resistance and *R*_0_ as the initial resistance without heating) and the temperature rise. The top TS and in situ TS both show the good linearity with high thermal sensing sensitivities of 0.26% °C^−1^ and 0.29% °C^−1^, respectively, which is comparable to previously reported flexible temperature sensors [29].

In order to reveal the heat-insulating performance of the ITTD with the G-HGM pad, the temperatures on both sides (unheating surface and heating surface) of the device are recorded by the top TS and in situ TS at different applied heating powers.  Figure 2(c) shows the temperature curves at the heating power of 220 mW for 300 s. The measured steady-state temperatures by the top TS and in situ TS are 51.6°C and 78°C, respectively, which are consistent with those (51.1°C and 77.7°C) measured by a thermal imager. As expected, the temperature on the unheating surface is much lower than that on the heating surface with a large steady-temperature difference of 26.4°C, which indicates a good heat-insulating effect. The temperature distribution on the heater is not uniform with the maximum and minimum temperatures as 78°C and 65°C, respectively, which could induce a nonuniform tissue ablation during the device operation. However, this may not be a major problem since the shape of the tumor is usually irregular. Moreover, the heater can be designed in a manner of nonequal width to achieve a uniform temperature distribution. The powers of 120 mW and 320 mW are also chosen to operate the heater with the steady-state temperature differences of 15.2°C and 40.1°C, respectively (Figure S4).  Figure 2(d) gives the steady-state temperature differences of the ITTD with the G-HGM pad at various applied heating powers. Furthermore, the heat-insulating performances of the ITTD with the PDMS pad and the G-PDMS pad are also investigated (Figure S5). The temperature difference between heating surface and the unheating surface increases with the heating power. To quantify the heat-insulating performance of the ITTD, we define the temperature reduction rate (TRR) as the relative steady temperature difference between the heating surface and the unheating surface over the steady temperature of heating surface. A larger TRR indicates a better heat-insulating performance.  Figure 2(e) shows the TRR as the function of the heating power for ITTDs with different pads. The TRR increases with the heating power. As expected, the G-HGM pad exhibits the best heat-insulating performance by combining the two strategies of increasing the interfacial thermal resistance and lowering the thermal conductivity. For example, the TTRs of PDMS and G-PDMS pads are only 22% and 30%, respectively, while the TTR of G-HGM can reach as high as 44% for the heating power of 320 mW.

In the process of the thermal therapy, the precise control of heating temperature is of great importance for improving the therapeutic effect and preventing thermal damage. The ITTD with the G-HGM pad is selected to demonstrate the precise temperature control capability. Five preset target temperatures (72.2°C, 72.9°C, 73.6°C, 72.9°C, and 72.2°C) of the heating surface with a small temperature difference of 0.7°C can be quickly reached by fine-tuning the applied heating power between 193.1 mW and 199.5 mW with the aid of in situ temperature sensing (Figure 2(f)). Thus, our ITTD could provide a closed loop heating with a precision of at least 0.7°C by implementing the in situ heater and temperature sensor.

### 2.3. In Vivo Experiment of Thermal Ablation for Antitumor

Thermal ablation is effective for local cancer treatment by increasing the temperature of the tumor (>50°C) to damage the tumor cells directly [30]. Although the antitumor effect by thermal treatment is remarkable [31], a lack of the real-time and in-situ temperature monitoring may cause the decrease of antitumor effect and the thermal damage of the normal tissues closed to the lesion area. Here, our ITTDs are implanted into the interface of subcutaneous tumor and skin of tumor-bearing mice (breast cancer cells, 4T1) to ablate the tumor while protect the normal skin tissue. Considering that all materials (e.g., polyimide and PDMS) and structures (e.g., PI/metal/PI layout) involved in our device have been widely used and proven to be safe in biointegrated electronics [32–34], we did not carry out the biocompatible test at this proof-of-concept stage. Despite the implantable device can make new risks and bring inconvenience, it also offers some benefits unreachable by other thermal therapeutic techniques, e.g., the implantable device may provide the tool to monitor the condition of tumor when integrated with other physiological sensors and operate as desired. The involving of transient materials in ITTD could further minimize the risks and inconvenience as the ITTD could dissolve with time. Moreover, as long as the side effects of open operation can be controlled with excellent thermal therapy performance, implantable thermal therapeutic devices are still desired in practical applications as demonstrated in implantable flexible drug release device for cancer treatment [24], implantable heater for cancer treatment [35], and implantable optoelectronic device for metronomic photodynamic cancer therapy [23].

**Figure 3:**
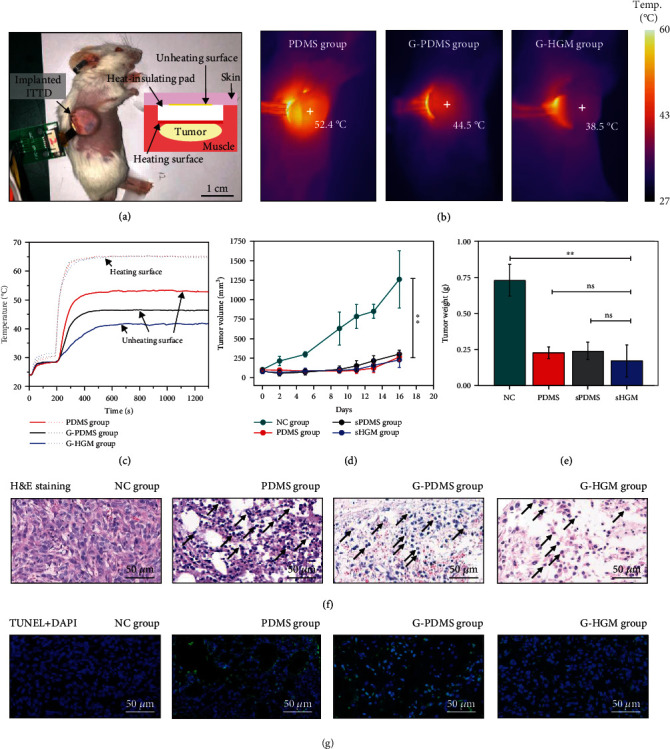
In vivo experiment of thermal ablation for antitumor. (a) Photo of the ITTD implanted in a tumor-bearing mouse. (b) Thermal images of the mouse skin in the treatment groups of the PDMS group (left), G-PDMS group (middle), and G-HGM group (right) during ablation. (c) Temperature curves measured by the top and in situ temperature sensors in the treatment groups. (d) Tumor volume curves of different groups (^∗∗^*P* < 0.01). (e) Tumor weight of different groups (^∗∗^*P* < 0.01) by day 16. (f) H&E staining images of the tumor tissues in different groups. (g) TUNEL and DAPI staining images of the tumor tissues in different groups. Blue: the fluorescence of DAPI; green: the fluorescence of TUNEL.

Figure 3(a) shows an anesthetized mouse undergoing thermal ablation with an illustration on the right to schematically show the ITTD implanted under the skin. The heating surface of the ITTD contacts the tumor while the skin is insulated by the heat-insulating pad. The in situ TS on the heating surface detects the temperature of the heater and tumor in real time; meanwhile, the top TS on the unheating surface records the temperature of skin for precaution of overheating. The tumor-bearing mice are randomly divided into four groups: (i) negative control group (NC, no treatment on the tumor), (ii) PDMS group (thermal ablation with the PDMS ITTD), (iii) G-PDMS group (thermal ablation with the G-PDMS ITTD), and (iv) G-HGM group (thermal ablation with the G-HGM ITTD). The depth of ablation can be controlled by the heating temperature and heating duration. All tumors in the treatment groups are heated at 65°C for 15 min followed by suturing and feeding.

The thermal imager gives the temperature profiles of the treatment groups, which shows that the skin temperatures directly above the hyperthermia region finally increase to 52.4°C, 44.5°C, and 38.5°C, respectively, for the PDMS group (Figure 3(b), left), G-PDMS group (Figure 3(b), middle), and G-HGM group (Figure 3(b), right). The results are consistent with the heat-insulating performances of these three types of pads. Figure 3(c) gives the real-time temperature curves from the in situ TS and the top TS. The dotted lines show the heating temperatures obtained from the in situ TS. In each treatment group, the heating temperature is controlled at 65°C for 15 min. The solid lines come from the top TS and represent the temperatures of the unheating surface (i.e., the normal skin tissue). There is an obvious difference in skin tissue temperature with 52.9°C, 46.4°C, and 41.8°C for the PDMS group, G-PDMS group, and G-HGM group, respectively. These results are slightly higher than those by the thermal imager since the top TS measured the temperature inside the skin. Only the G-HGM group gives a temperature below the critical temperature (43°C) for thermal damage of tissue, which indicates that the G-HGM group shows the best heat-insulating effect and induces no thermal damage at all, while the other two groups will cause a certain thermal damage during thermal ablation.

The tumor volume changing with the number of days are measured to assess the antitumor therapeutic effect of the device (Figure 3(d)). The initial tumor volume is around 100 mm^3^ in all groups before treatments. No distinct tumor volume increment in the treatment groups can be observed in the first 9 days while tumor volume increases to 595 mm^3^ in the NC group, indicating the robust antitumor performance of the ITTD with the tumor inhibition rates of 84.8%, 76.9%, and 77.6% for the PDMS group, G-PDMS group, and G-HGM group, respectively. Thereafter, the tumor begins to recrudesce because of residual tumor cells, but the growth speed of the treatment group is much less than that of the NC group. Sixteen days after the treatment, mice are sacrificed for weight statistics and pathological biopsies observation of tumors. Figure 3(e) shows the tumor weight decreases from 0.73 g in the NC group to 0.23 g, 0.24 g, and 0.17 g, respectively, in the PDMS, G-PDMS, and G-HGM groups.

The histology of tumor tissue by day 16 is assessed by hematoxylin and eosin (H&E) staining (Figure 3(f)). Intact tumor tissue can be found in the NC group, while obvious necrotic regions and damaged cells are observed in the treatment groups. As arrows show, nuclei of some tumor cells were pyknotic and fragmented with deeper color in the PDMS, G-PDMS and G-HGM groups. Terminal deoxynucleotidyl transferase deoxynucleotidyl triphosphate nick end labelling (TUNEL), which could detect fractured DNA strand associated with apoptosis, is performed to investigate apoptotic cells in tumor area (Figure 3(g)). The quantity of TUNEL-positive cells increase significantly in the treatment groups compared with the NC group. These results illustrate the outstanding tumor inhibitory effect of thermal therapy. Furthermore, intact histology can be found in the H&E slices of the heart, liver, spleen, lung, and kidney of each group, indicating the negligible side effect from thermal treatment (Figure S6). There is no obvious change in the bodyweight of mice in in vivo experiment, reflecting the biosafety of the device (Figure S7).

### 2.4. Thermal Protection of the Normal Tissue Adjacent to the Tumor

**Figure 4:**
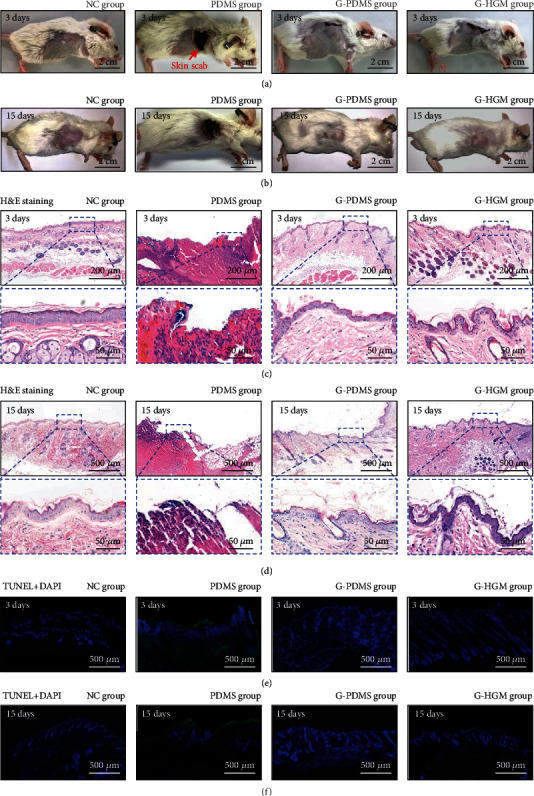
Thermal protection of the normal tissue adjacent to the tumor. (a, b) Photos of the skin tissues around tumor in different groups by day 3 and day 15. (c, d) H&E staining images of the skin tissues around tumor in different groups by day 3 and day 15. (e, f) TUNEL and DAPI staining images of the skin tissues around tumor in different groups by day 3 and day 15. Blue: the fluorescence of DAPI; green: the fluorescence of TUNEL.

Although the normal tissues can survive a higher temperature than that of tumor (4-5°C), they are still susceptible to thermal damage during thermotherapy without thermal protection. Despite all treatment groups can achieve excellent antitumor effects, the levels of thermal damage to the normal tissue adjacent to tumor are quite different due to the distinct heat-insulating performance of pads. The normal tissue should be protected since a damaged tissue is not only difficult to maintain body fluids and keep electrolyte balance but also susceptible to bacteria and other pathogens, resulting in severe infection [36]. As shown in Figure 3(c), the skin temperature of the PDMS group is the highest (52.9°C), which will cause irreversible thermal damage [37]. Figures 4(a) and 4(b) show the optical images of the ablation region of the NC group, the PDMS group, the G-PDMS group, and the G-HGM group after 3 days and 15 days, respectively. A severe thermal damage to the skin is observed in the PDMS group due to the excessive heating, while no obvious thermal damages are observed in the G-PDMS and G-HGM groups. Notably, the surgical sutures degrade and the wound completely heal by day 15, indicating the negligible side effect in the treatment.

To further investigate the thermal effect on the normal tissue, the detailed pathological examination of the skin around the tumor is evaluated. H&E staining of skin tissue biopsies is used to analyze the skin structure after 3 days (Figure 4(c)) and 15 days (Figure 4(d)). In the PDMS group, the skin suffers irreversible third-degree burn damage with the destructions of the epidermis and dermis, while the structure of the skins in the G-PDMS and G-HGM groups remain intact and no obvious change of the epidermal thickness can be observed. Specifically, the G-HGM group shows more cutaneous appendages (sebaceous gland and apocrine sweat gland) by day 3 than the G-PDMS group due to the better thermal protection of G-HGM pad. TUNEL staining and DAPI staining are executed to assess the skin damage after 3 days (Figure 4(e)) and 15 days (Figure 4(f)) for assessing the status of apoptosis. The PDMS group shows an evident TUNEL signal, while the G-PDMS and G-HGM groups have no distinct homologous signals, indicating a little impairment. These results suggest that the heat-insulating design of the device enable the effective skin protection when heating the tumor.

## 3. Discussion

In summary, we report an implantable thermal therapeutic device (ITTD) with precise temperature control capability enabled by a multifunctional foldable electronic device wrapped on a heat-insulating composite pad to optimize the therapeutic effect on the tumor while provide thermal protection to the normal tissue. Systematic experimental studies are carried out to investigate the heating, monitoring, and heat-insulating performances of the ITTD. The heater exhibits a good linearity with the applied power in a broad heating range up to 102°C. The heat-insulating composite pad can yield a temperature reduction rate of 43.5% for the heating power of 320 mW. It is shown that the proposed ITTD could provide a precise temperature control with the precision of at least 0.7°C by implementing the in situ heater and temperature sensor. Demonstration of the ITTD for tumor ablation illustrates that this device is capable of monitoring and adjusting ablation temperature precisely in real time, detecting the adjacent tissue's temperature, and providing effective thermal protection. This proof-of-concept research creates a promising route to develop ITTD with precise temperature control capability, which is highly desired in practical applications of tumor ablation, wound management, drug release, and other pathological diagnoses and treatments.

## 4. Materials and Methods

### 4.1. Fabrication of the Heat-Insulating Pad

Cylindrical and grooved aluminum alloy molds were prepared for the heat-insulating pads. The PDMS (Sylgard 184, Dow Corning) mixture was prepared with the base: crosslinker mass ratio of 10 : 1. The PDMS pad and the G-PDMS pad were fabricated by casting the PDMS mixture in different molds, followed by baking for 2 h at 80°C. As for the G-HGM pad, the PDMS mixture was mixed with hollow glass microspheres at the mass ratio of 5 : 1 by manually stirring for 10 min. The resulting composite mixture was then casted in the grooved mold, followed by baking for 2 h at 80°C.

### 4.2. ITTD Fabrication

The ITTD is composed of a multifunctional foldable thin film electronic device and a heat-insulating pad. To fabricate the foldable thin film electronic device, a glass substrate was cleaned and then baked at 120°C for 15 min on a hotplate for dehydration. The PI precursor solution (ZKPI-305IIE, POME) was spin-coated (2500 rpm for 40 s, 7 *μ*m) on the substrate, followed by curing at 230°C for 2.5 h. The serpentine metal layer (Cr/Au, 5/150 nm) was patterned on the PI through photolithography and electron beam evaporation (DZS-500, SKY). Another PI layer as encapsulation was spin-coated (2500 rpm for 40 s, 7 *μ*m) on it and patterned by an inductively coupled plasma etching (ICP-100A, TAILONG). The foldable thin film electronic device was then released in the buffer oxidation etching solution (BOE, RESEMI) and picked up by a water-soluble tape (ASW-1, AQUASOL) exposed on its underside for deposition of a thin layer of Cr/SiO2 (5/30 nm) by electron beam evaporation. The foldable thin film electronic device was then folded onto the heat-insulating pad with its surface activated by ozone to complete the fabrication of the ITTD.

### 4.3. FEA Bending Analysis

Three-dimensional (3D) finite element models were established in ABAQUS to investigate the strain distributions of the foldable electronic device subjected to a bending moment. The device consisted of a layer of serpentine metal (chromium/gold) trace sandwiched by the patterned PI layers. The thicknesses of chromium, gold, and PI are 5 nm, 150 nm, and 14 *μ*m, respectively. The chromium layer was neglected in the finite element model due to its extremely small thickness. The elastic moduli of PI and gold are 2.5 GPa and 77.2 GPa, respectively. Poisson's ratios of PI and gold are 0.34 and 0.44, respectively. The in situ senor was fixed, and a moment of 2.1 × 10^−6^ N·m was applied to the top sensor to make the device bend.

### 4.4. Experimental Animal/Surgical Procedures

5 × 10^4^ 4T1 cells were injected into the fat pad of Balb/c mice (~20 g) to establish orthotopic breast cancer. When the tumor volume developed to 100 mm^3^, these mice were divided randomly into four groups: (i) negative control group, (ii) PDMS group, (ii) G-PDMS group, and (iv) G-HGM group. A tiny wound was performed on the side of breast cancer after anesthetization. Thereafter, PDMS, G-PDMS, or G-HGM ITTD was carefully implanted to the interface between the skin and subcutaneous tumor. The incision was closed with sutures so that the ITTD is tightly sandwiched between the skin and the tumor, thus avoiding the device movement. The power was controlled to maintain the temperature of heater at 65°C for 15 minutes. The tumor volume and body weight were recorded twice a week. On the 3rd and 15th days, a mouse in each group was selected randomly and sacrificed. Tumor, the skin around cancer, and other organs (the heart, liver, spleen, lung, and kidney) were collected for the following assay. On 16th day, all mice were sacrificed. Tumors were taken out for photographing and weighing. Tumor tissues and other organs were immersed in 4% paraformaldehyde for 24 hours and dehydrated with different concentrations of alcohol. Dehydrated tissues were embedded in paraffin and whittled to five-micron slices by microtome. These slices were stained by hematoxylin and eosin and observed by microscope for the H&E assay. Similarly, they were treated with Proteinase K working solution and TUNEL reaction solution and then analyzed by confocal laser scanning microscopy (Ex/Em = 490 nm/520 nm) for TUNEL assay.

### 4.5. Statement for Using Experimental Animals

The animal use protocol has been reviewed and approved by the Institutional Animal Care and Use Committee (approval no. ZJCLA-IACUC-20020030).

## Data Availability

All study data are included in the article and/or supporting information.

## Supplementary Materials

Supplementary Materials Figure S1: schematic fabrication process of the multifunctional foldable electronic device. Figure S2: schematic fabrication process of the grooved heat-insulating pad. Figure S3: the thermal conductivity of PDMS composite pads doped with 0-20% *w*/*w* HGM. Figure S4: temperature curves of the heating surface and the unheating surface of the ITTD at applied heating powers of (a) 120 mW and (b) 320 mW. Figure S5: influences of the power on the heat-insulating performance of (a) PDMS ITTD and (b) G-PDMS ITTD. Figure S6: H&E slices of vital organs of groups of NC, PDMS, G-PDMS, and G-HGM by day 15. (a) Heart. (b) Liver. (c) Spleen. (d) Lung. (e) Kidney. Figure S7: body weight changes with the days.
