# Supplementary material for: Implantable Thermal Therapeutic Device with Precise Temperature Control Enabled by Foldable Electronics and Heat-Insulating Pads
Source: Research (Wash D C). 2022 May 24;2022:9787296. doi: 10.34133/2022/9787296 (PMC9161283; doi:10.34133/2022/9787296)
Supplement: Supplementary Materials — Figure S1: schematic fabrication process of the multifunctional foldable electronic device. Figure S2: schematic fabrication process of the grooved heat-insulating pad. Figure S3: the thermal conductivity of PDMS composite pads doped with 0-20% w/w HGM. Figure S4: temperature curves of the heating surface and the unheating surface of the ITTD at applied heating powers of (a) 120 mW and (b) 320 mW. Figure S5: influences of the power on the heat-insulating performance of (a) PDMS ITTD and (b) G-PDMS ITTD. Figure S6: H&E slices of vital organs of groups of NC, PDMS, G-PDMS, and G-HGM by day 15. (a) Heart. (b) Liver. (c) Spleen. (d) Lung. (e) Kidney. Figure S7: body weight changes with the days. [file 9787296.f1.docx]

Supporting Information

**Implantable Thermal Therapeutic Device with Precise Temperature Control Enabled by Foldable Electronics and Heat-Insulating Pads**

Min Cai^1^†, Huang Yang^2^†, Liyin Shen^2^, Shuang Nie^1^, Zhengwei Mao^2^, Changyou Gao^2^*, Yang Zhu^2^* & Jizhou Song^1,3^*

^1^Department of Engineering Mechanics, Soft Matter Research Center, and Key Laboratory of Soft Machines and Smart Devices of Zhejiang Province, Zhejiang University, Hangzhou 310027, China

^2^MOE Key Laboratory of Macromolecular Synthesis and Functionalization, Department of Polymer Science and Engineering, Zhejiang University, Hangzhou 310027, China

^3^State Key Laboratory of Fluid Power and Mechatronic Systems, Zhejiang University, Hangzhou 310027, China

†M.C. and H.Y. contributed equally to this work.

* Corresponding author. Email:cygao@zju.edu.cn; zhuyang@zju.edu.cn; jzsong@zju.edu.cn

**Figure S1**


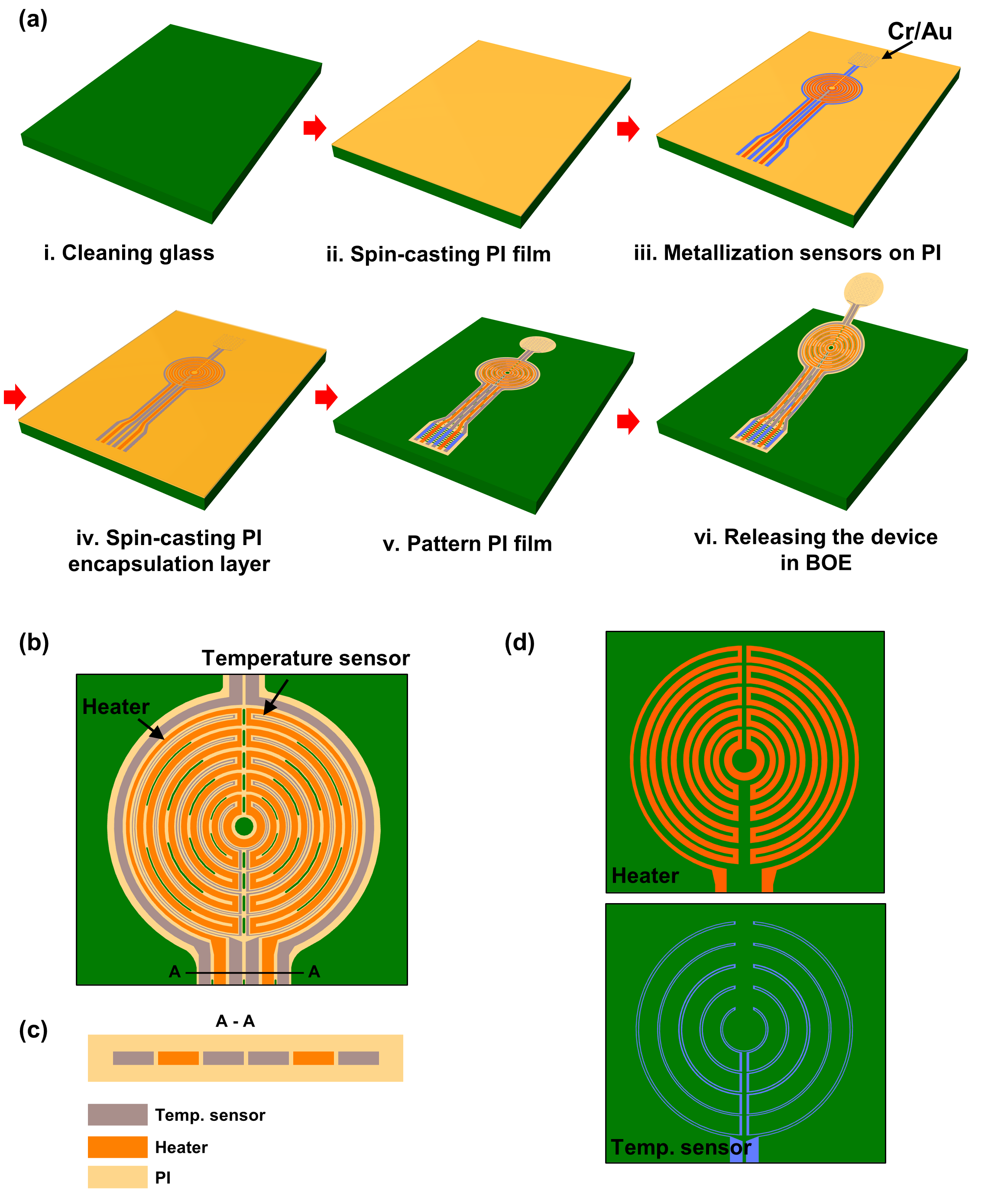


**Figure S1**. Schematic fabrication process of the multifunctional foldable electronic device. (a) Perspective views of the fabrication process. (b) Structural design of the heating region with an in-situ heater/temperature sensor, which shares the same pattern but different geometric dimension. (c) Cross-section views of the device. (d) Construction schematics of separate heater (*Top*) and in-situ temperature sensor (*Bottom*).

**Figure S2**


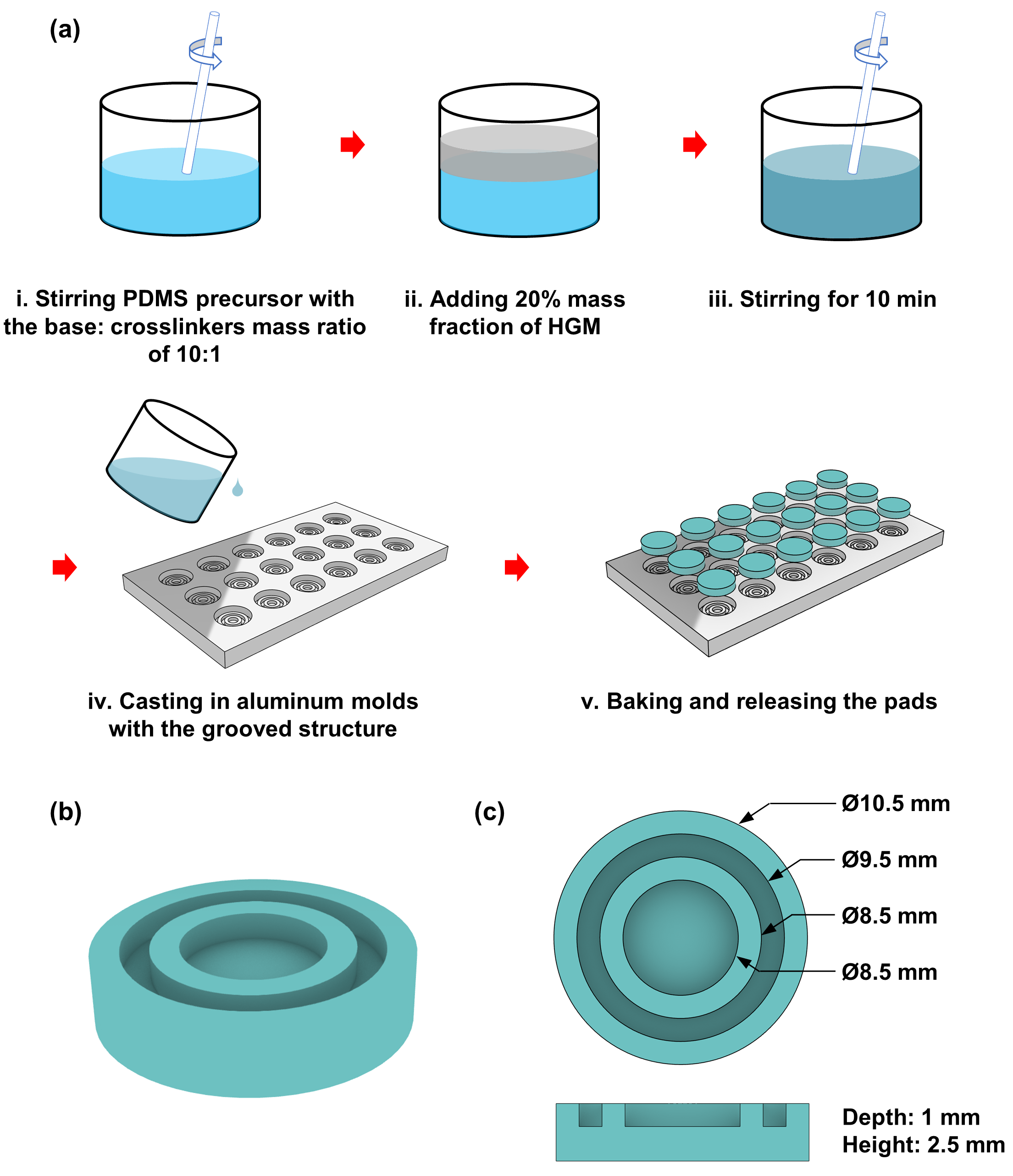


**Figure S2.** Schematic fabrication process of the grooved heat-insulating pad. (a) Perspective views of the fabrication process. (b) Schematic of the G-HGM. (c) Dimensional drawing. G-PDMS and G-HGM have the same structure.

**Figure S3**


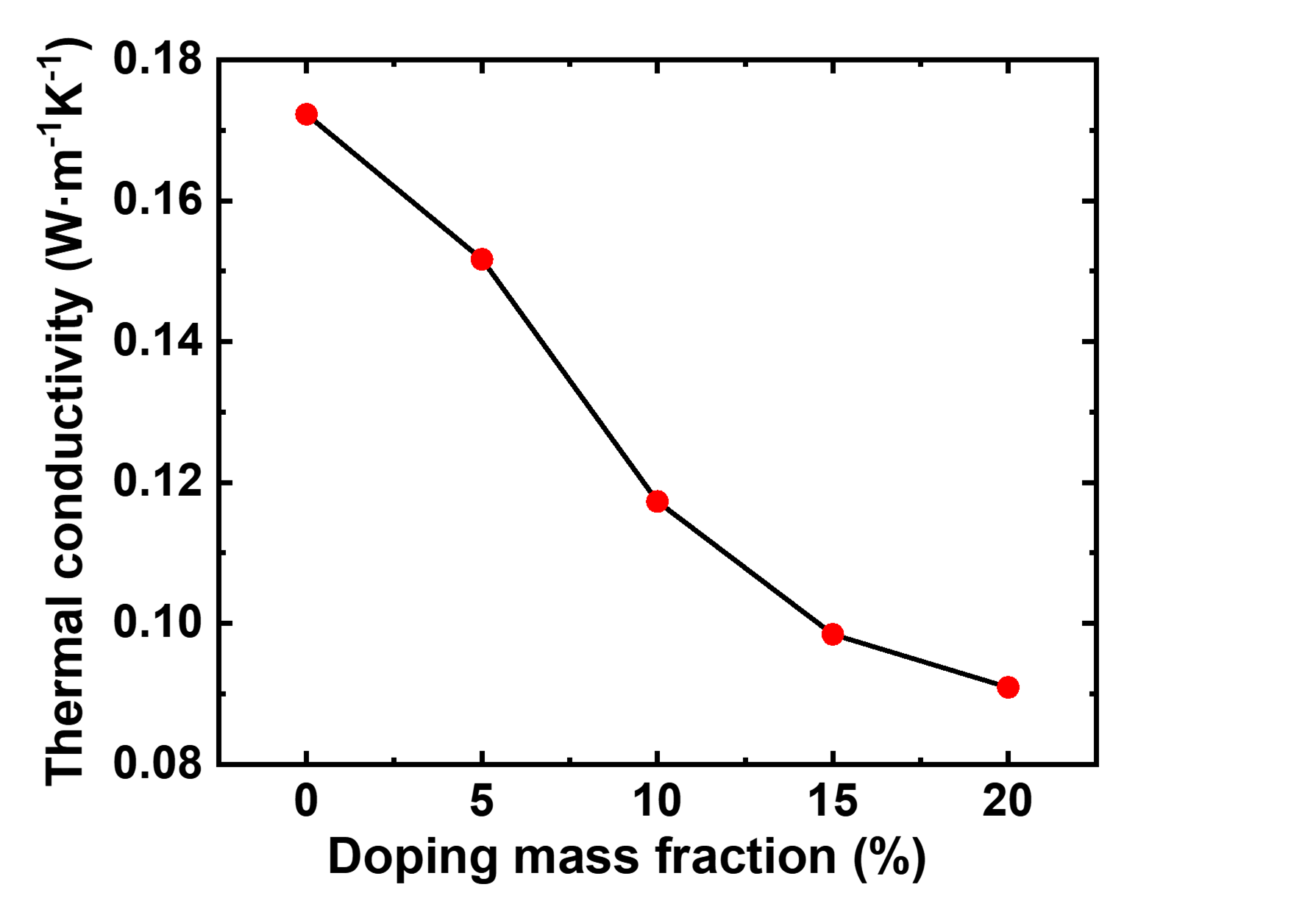


**Figure S3.** The thermal conductivity of PDMS composite pads doped with 0-20% w/w HGM.

**Figure S4**


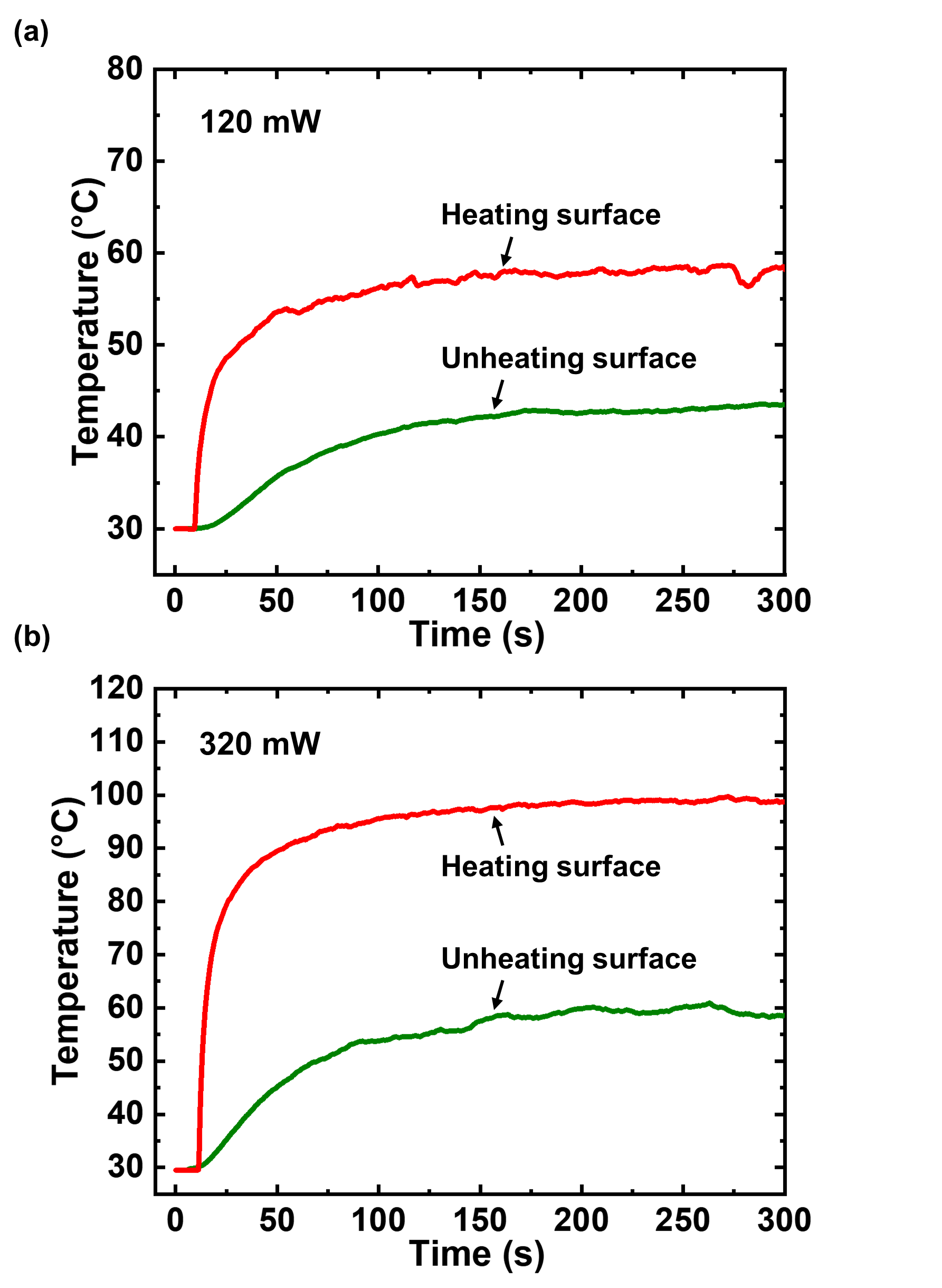


**Figure S4.** Temperature curves of the heating surface and the unheating surface of the ITTD at applied heating powers of (a) 120 mW and (b) 320 mW.

**Figure S5**


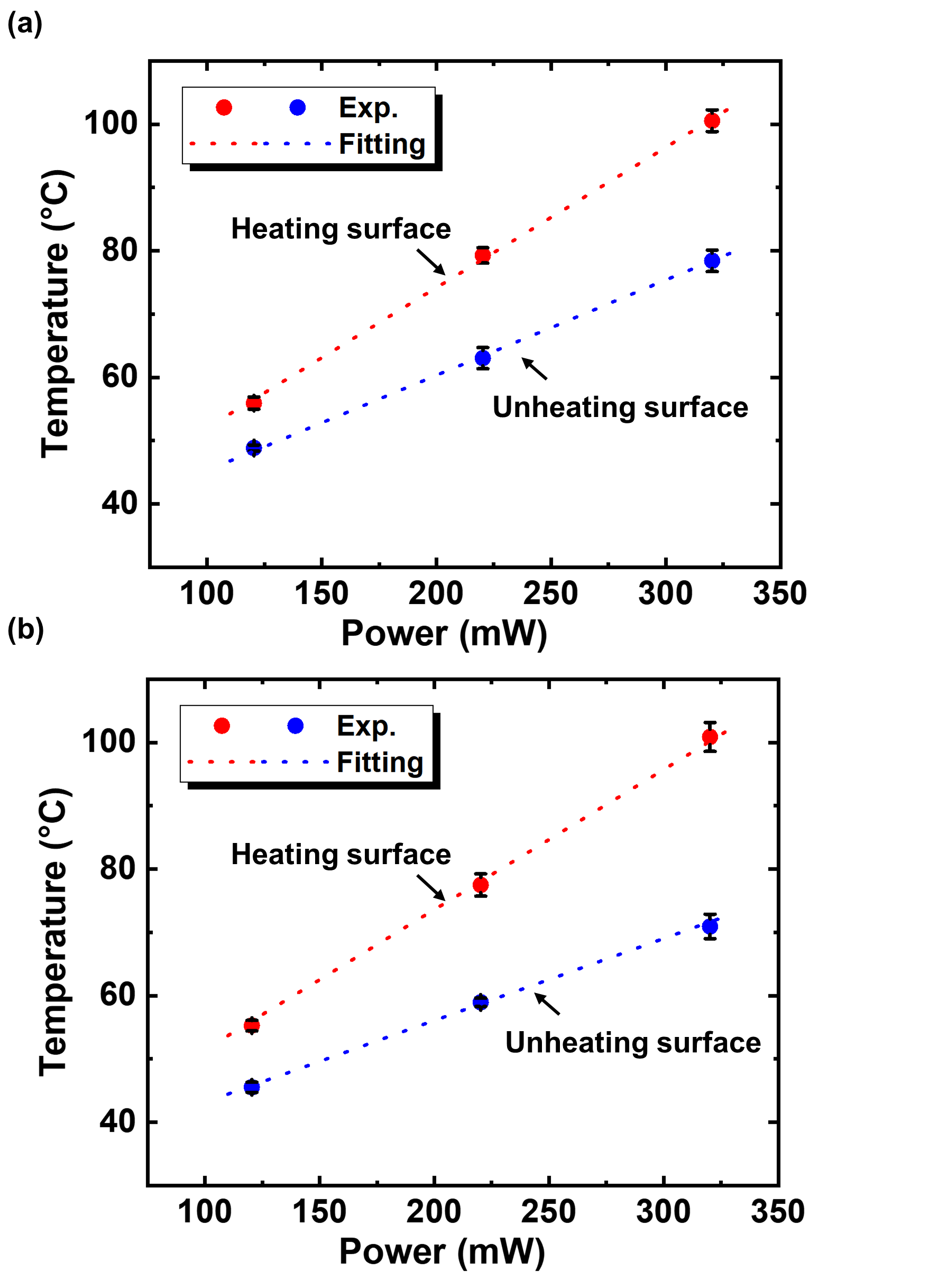


**Figure S5.** Influences of the power on the heat-insulating performance of (a) PDMS ITTD and (b) G-PDMS ITTD.

**Figure S6**


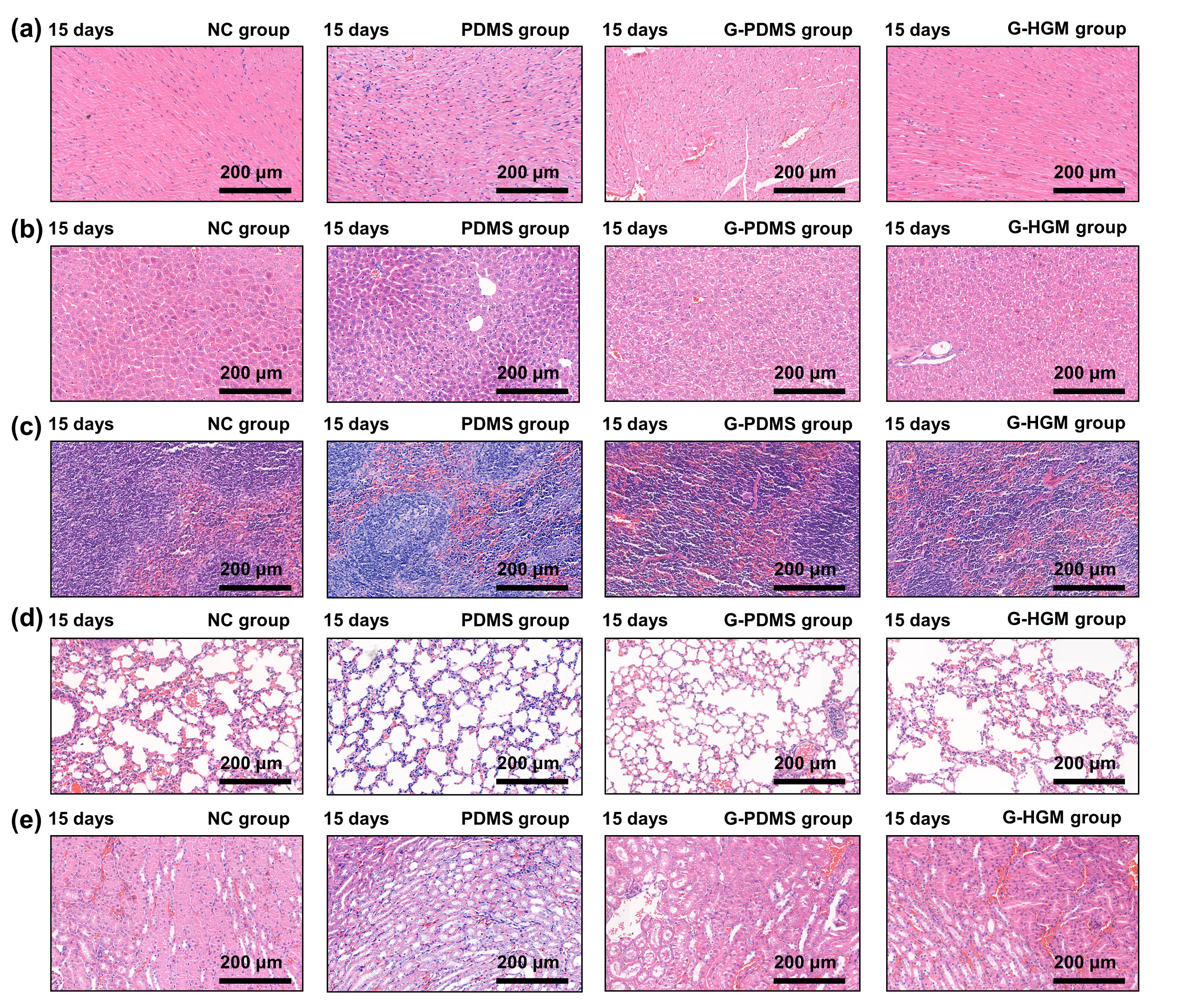


**Figure S6.** H&E slices of vital organs of groups of NC, PDMS, G-PDMS, and G-HGM by day 15. (a) Heart. (b) Liver. (c) Spleen. (d) Lung. (e) Kidney.

**Figure S7**


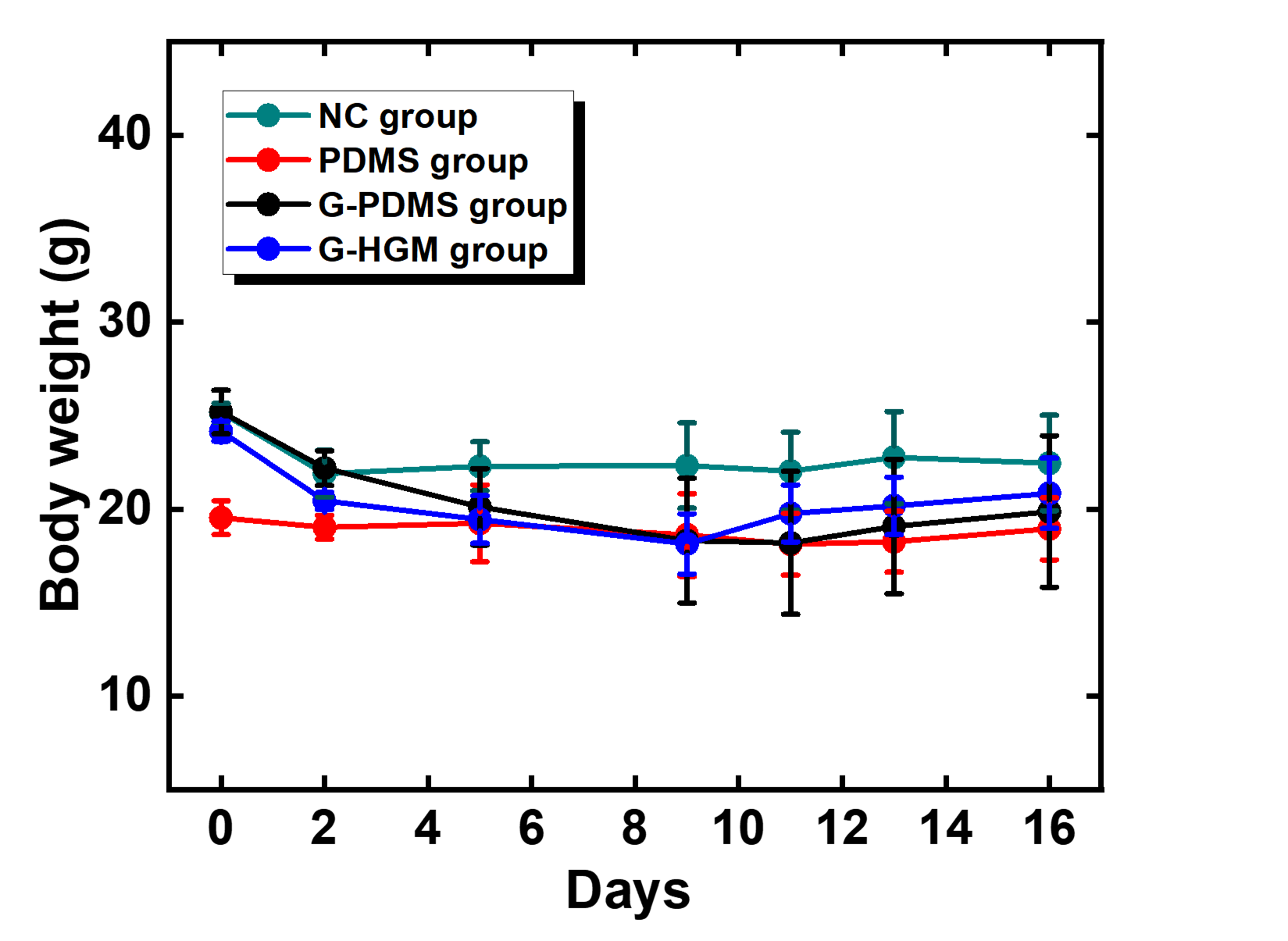


**Figure S7.** Body weight changes with the days.
